# The Therapeutic Approaches Dealing with Malocclusion Type III—Narrative Review

**DOI:** 10.3390/life15060840

**Published:** 2025-05-22

**Authors:** Zdenka Stojanovic, Nadica Đorđević, Marija Bubalo, Milos Stepovic, Nemanja Rancic, Miroslav Misovic, Milka Gardasevic, Maja Vulovic, Ivana Zivanovic Macuzic, Vesna Rosic, Nikola Vunjak, Simonida Delic, Kristijan Jovanovic, Melanija Tepavcevic, Ivona Marinkovic, Zlata Rajkovic Pavlovic

**Affiliations:** 1 Medical Faculty of the Military Medical Academy, University of Defence, 11000 Belgrade, Serbia; alexandarst98@gmail.com (Z.S.); nece84@hotmail.com (N.R.); miki_misic@yahoo.com (M.M.); 2 Department of Dentistry, Faculty of Medicine, University of Pristina in Kosovska Mitrovica, 38220 Kosovska Mitrovica, Serbia; 3 Department of Anatomy, Faculty of Medical Sciences, University of Kragujevac, 34000 Kragujevac, Serbia; 4 Institute for Radiology, Military Medical Academy, 11000 Belgrade, Serbia; 5 Clinic for Maxillofacial Surgery, Military Medical Academy, 11000 Belgrade, Serbia; 6 Department of Histology and Embryology, Faculty of Medical Sciences, University of Kragujevac, 34000 Kragujevac, Serbia; vecarosic@gmail.com; 7 Faculty of Medical Sciences, University of Kragujevac, 34000 Kragujevac, Serbia; 8 Department of Dentistry, Faculty of Medical Sciences, University of Kragujevac, 34000 Kragujevac, Serbia; zlatakg@yahoo.com

**Keywords:** malocclusion class III, prognathism, mandibular prognatism, malocclusion diagnosis, therapy of class III, cephalometric analysis

## Abstract

According to the World Health Organization, malocclusion type III is third, most important oral health problem. It may be the least prevalent malocclusion, but it is the most noticeable and challenging for orthodontic therapy. With this narrative review, we wanted to give a summation of the most current knowledge about diagnostics, different therapy options, limitations, and additional factors that can influence the therapy of class III malocclusion to help clinicians and researchers focus on the specific approaches. Therapy options were divided into the following groups: orthopedic, orthodontic, and surgical. The SNA, SNB, and ANB angles (cephalometric values) are the best ones to examine improvements in different skeletal improvements, while dentoalveolar improvements were also described, emphasizing the limitation of orthopedic therapy to change the skeletal discrepancy. Eruption-guided appliances and chin cups are more effective in early childhood, mixed dentition, during permanent teeth eruption, with no significant skeletal discrepancy. If a discrepancy exists, a face mask is the first therapy choice. The therapy of an underdeveloped maxilla can be solved with different palate expanders. Bulkiness, lack of long-term results, and duration of therapy make fixed orthodontic appliances with braces and elastic traction favorable nowadays. If the skeletal discrepancy is major, a surgical approach should be considered. One of the main limitations in articles is the combination of different therapy approaches, ages, and dentition preferred for device application, duration of therapy, and lack of information about long-term outcomes. On the other hand, the lack of original articles is noticeable, so further research should be done.

## 1. Introduction

Contacts between the teeth of the upper and lower jaw that provide normal function of the orofacial system (as breathing, chewing, swallowing, speaking) represent occlusion. Deviations in the occlusal relationship between teeth are defined as malocclusion. Still, the ideal teeth position is managed according to race, facial profile, facial balance, and esthetic concerns [1]. The father of modern orthodontics, Dr. Edward Harthley Angle, defined three types of malocclusion as a result of the intercuspidal relationship between maxillary and mandibular first permanent molars [2]. While 92% of cases of malocclusion have an unknown cause, likely because of a long-term interaction between genetic and environmental factors, etiological factors are still roughly classified as prenatal and postnatal. Also, it must be noted that physiological adaptation in the inclination of the teeth can mimic the main factor trying to functionally compensate for skeletal discrepancy [3].

Each factor has a contributing effect, which aggravates the characteristics of a malocclusion. The variety of clinical presentation of malocclusion may affect oral health, make teeth brushing difficult, and increase the risk of caries, trauma, and oral diseases. Also, the malposition of teeth is an etiological factor in periodontal disease [3,4].

A class III molar relationship presents a mandible positioned anterior to the maxilla and mandibular teeth protrude over the maxillary teeth. Class III malocclusion is distinguished by the alignment of teeth into three types. In class 3 type 1, the arch is abnormally shaped. In class 3 type 2, the mandibular teeth are tilted lingually. In class 3 type 3, the maxillary teeth are tilted lingually [5]. Different types of this malocclusion have varying causes, severity, and treatment approaches [6,7,8].

### 1.1. Prevalence and Etiology of Class III

According to the World Health Organization, malocclusion type III is the third, most important oral health problem, behind dental caries and periodontal diseases [9]. The class III malocclusion may be the least prevalent malocclusion, but it is the most noticeable and challenging for orthodontic therapy, affecting facial esthetics and quality of life [10].

The genetic predisposition is the most intriguing factor for scientists, and mandibular prognathism has been linked to chromosomes 1p36, 6q25, and 19p13.2, but ethnicity has also proven to be a predisposing factor [11]. From the information from systematic reviews, considering the ethnicity, the lowest prevalence is among Caucasians and highest among Asians. In other articles dealing with prevalence, the lowest was among the European population (0.48 to 4.0%), and higher in African population (3.0 to 8.0%) and Mexicans (8.3%). Looking by the country, it is noted that Italy, Nigeria, and Jordan are the least prevalent (all combined, roughly 2.4%), while Puerto Rico, Saudi Arabia and Brazil are the most prevalent (all combined, roughly 16.3%) [12,13].

Several syndromes are related to the class III malocclusion, where those midface components were affected in Apert and Down Syndromes, lower face in Klinefelter Syndrome, and midface and lower face components in X-linked hypohidrotic ectodermal dysplasia [14]. Malocclusion III was also related with osteogenesis imperfect, but in fewer amounts than other skeletal class abnormalities [15].

The functional part of etiology of malocclusion type III considers cranio-cervical posture where cervical inclination seems to be higher in the patients with class III, with limitations in breathing due to deviated septum, or disease where the breathing is dominantly through the mouth, but also from bad habits as the posture of the tongue and chewing habits [16,17,18,19]. Endocrinological problems may cause the predominant growth of distal bones, including the mandible. Certain medications (combination of thyroxine and prostaglandin E2) used in therapy may help in growth arrest of the mandible, as per the results of experimental study [20].

The growth model of the cranial base is essential for the development of malocclusions, among other things, because the sagittal position of the upper and lower jaws depends on the developmental changes in the anterior cranial base. Observed in relation to the cranial base, during growth, the mandible moves translatatory downward and forward, while simultaneously increasing its size by growing backward and upward, thus maintaining constant contact with the cranium. During prenatal and early postnatal development, the growth of the mandible (viscerocranium) is less intense than the growth of the cranial base (neurocranium). The bones of the maxillary mass, although part of the viscerocranium, have growth dynamics more similar to the growth of the bones of the cranial base due to their direct, sutural connection with them. Thus, at the age of 6, the cranial base has largely completed its growth, while the growth of the mandible has only gained full intensity from the age of 5 to 6 and continues for the next 10 years [21,22,23,24].

The skeletal class III malocclusion phenotype is diverse and typically defined by maxillary retrusion, mandibular protrusion, or both. The true class III malocclusion which is characterized by the mandibular prognathism is a not so common version of class III, and roughly 40% of all malocclusion class III are actually caused by maxillary retrognathism. Also, there are mixed types of both maxillary retrognathism and mandibulary prognathism, or pseudo prognathism (maxillary retrognathism and mandibular normognathism) [11].

### 1.2. Diagnosis of Malocclusions

**Figure 1:**
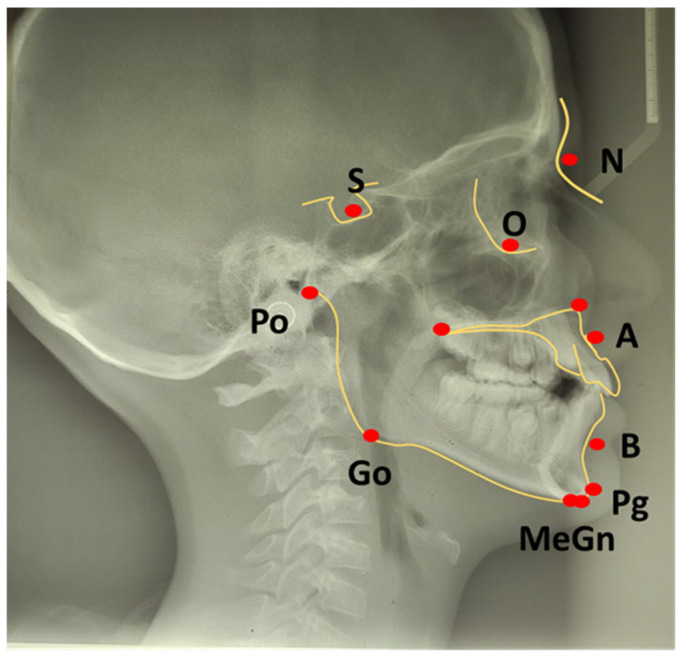
Points for cephalometric measurements. Nasion—the most anterior part of frontonasal suture (N), Sella turcica—central point (S), A point—the deepest point of contour of the premaxilla (A), B point—the deepest point of contour of the mandible (B), Gonion—point that creates crossing line of the inferior and posterior border of mandible (Go), Menton—the lower point of mandible symphysis (Me), Pogonion—the most anterior point of mandible (Pg), Gnathion—Midpoint of pogonion and menton (Gn), Porion (Po) and Infraorital (O).

Evaluation of malocclusion starts with clinical diagnosis by pediatric dentists and orthodontists [25]. At the same time, further skeletal discrepancies, relations among the maxilla and mandible, deviation of the temporomandibular joint, and other potential anatomic anomalies must be provided using radiographic examination. A panoramic radiograph can detect mandibular asymmetry, differences in the shape and positions of the right and left condyles, supernumerary or missing teeth, mandibular deviation and ramus height and width differences [26]. Also, the standard cephalometric assessment is based on a 2D scan, either sagittal (lateral) or coronal (posteroanterior) where using drawn lines, multiple landmarks and angles can determine the vertical and horizontal relationship of face, scull and jaws. Cephalometric analysis is a deductive method used for diagnosis and therapy planning, which also provides quantification of facial growth and analysis of transversal dimension and facial asymmetry (Figure 1).

Further improvement in the analysis of craniofacial structures provided non-invasive 3D imaging techniques as computed-tomography (CT), cone-beam CT (CBCT), and magnetic resonance imaging (MRI). The advantages in the scanning system avoid the head fixation and prevent structure distortion and magnification, allowing the full assessment of all anatomic structures in all planes in preoperative evaluation, surgical planning, and further inter-occlusal relationship [27]. CBCT has become the main 3D diagnostics method in all branches of dentistry. It can also be useful for analysis of TMJ and patients’ airway, also in reconstruction planning of clefts. Lately, the utilization of artificial intelligence (AI), with special machine learning algorithms, is becoming more common [28]. This method is applied in orthodontic diagnosis, such as cephalometric analysis and calculating skeletal-maturation-stage, treatment planning, and outcome prediction, especially in the diagnosis and treatment of angle class III malocclusions [29].

Evaluation and classification of malocclusion was given by Steiner analysis that estimates skeletal, dental, and soft tissue analyses using different points, planes, and angles in a two-dimensional profile cephalometric radiograph scan (Table 1) [30].

Skeletal analysis gives information of the position of the maxilla and mandible to the cranial base, the mutual relationship between the upper and lower jaw and the occlusal relationship to the cranium.

**Figure 2:**
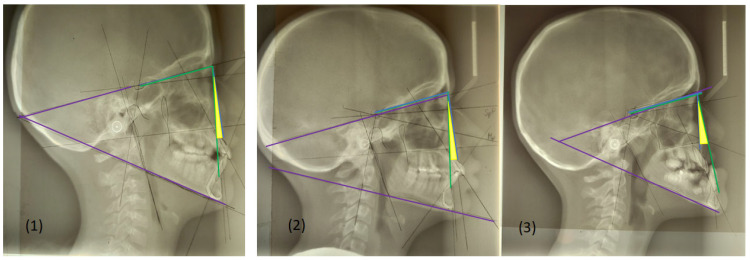
According to the Angle classification, class I is described as normocclusion with cephalometric angles approximately SNA = 82°, SNB = 80°, and ANB = 2°, class II with values SNA ≥ 82°, SNB ≤ 80°, ANB > 2 and class III SNA ≤ 82°, SNB ≥ 80°, and ANB < 2°.

- SNA angle is formed between points of center sella turcica (S) and nasion (N) as the most anterior point of frontonasal suture, and subspinal point of deepest spot of contour of premaxiila (A), which presents the position of maxilla anteriorly or posteriorly to the cranial base. An average value of SNA angle is 80° ± 2°, while a higher value shows that the maxilla is protrusive, and a lower value indicates that the maxilla is more retrusive than normal;
- SNB angle is formed by connecting points of center sella turcica (S) and nasion (N) as the most anterior point of frontonasal suture, and the point that presents the deepest spot of the mandible (B). The average value of SNB is 78° ± 2°. A value above indicates that the mandible is more anterior to the cranial base, or protrusive, and a value below is a more backward, retrusive position;
- ANB angle is the angle between point A-N-B or the difference between SNA and SNB angle, with a normal discrepancy between maxilla and mandible ± 2°. A higher value points to the angle class II malocclusion relationship, while a lower angle indicates the angle class III malocclusion (Figure 2).

The normal value of angles varies depending on race.

The mandibular plane angle forms the mandibular plane (line of inferior border of gonion (Go) to gnathion (Gn)) and SN line, the mean is 32°. Mandibular plane is important for understanding the jaw growth pattern, so if the mandibular plane angle is higher than 32° than it shows a vertical growth pattern, while a lower value suggests horizontal growth.

**Figure 3:**
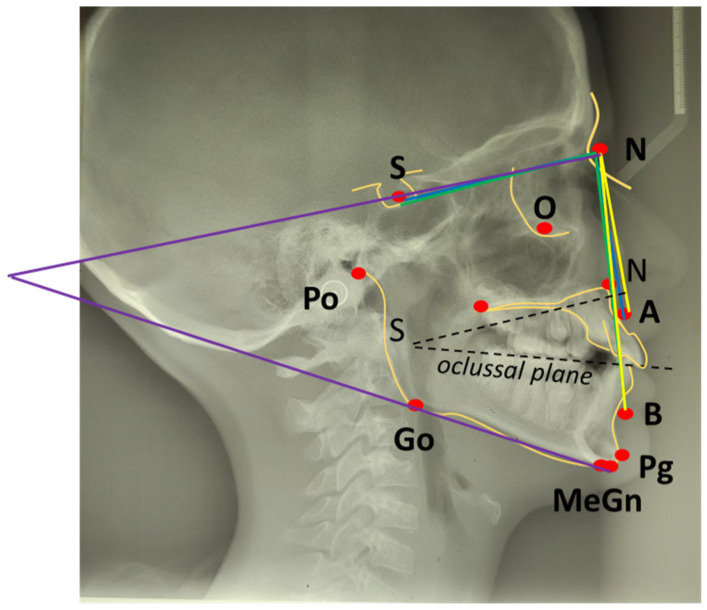
Skeletal measurements of cephalometric analysis: SNA angle, SNB angle, ANB angle, Mandibular plane angle, Occlusal plane angle.

The occlusal plane crosses through the cusps of the first premolars and molars. An angle is created between the occlusal plane and SN plane, and the average is 14°. An occlusal plane angle value more than 14° suggests long face, vertical growth, and skeletal open bite, while less than 14° is connected with horizontal growth and deep bite [31]. These measurement points are analysed in different therapeutic approaches for malocclusion class III with an aim of finding the most effective method of therapy (Figure 3).

Other denotoalveolar and temporomandibular structures can affect the therapy of class III malocclusion. The collum angle, tooth dimensions, root length, and alveolar bone thickness have a significant impact on orthodontic diagnosis and therapy planning and, in the study conducted by Rawaqa, class III subjects had an increased root length of the upper first premolars, upper central incisors, and lower first and second premolars compared to other skeletal classes [32].

#### Diagnosis of Malocclusion Type III

1. According to study from 2015 significant measurements in diagnosis of skeletal class III are: mandibular prognathism by the sagittal position of the mandible to the anterior cranial base (SNB);
2. Prognathism by the anterior sagittal position of the symphysis mentalis to the anterior cranial base plane;
3. Mandible hyperdivergent growth;
4. Retroinclination of lower teeth;
5. Occlusal plane tilting towards the mandible basis;
6. Anterior position of TMJ;
7. Posterior condylar growth, with the opening of the mandibular angle;
8. Facial anterior height growth.

Also, the parameters of cranial base shortening, underdeveloped maxilla, upper central incisor proclination, and facial deficiency of the zygomatic region can be noticed. In prepuberty ages (under 14 years), parameters are established throughout the skeletal growth, while puberty (over the age of 14) is significant in maximum mandibular length growth, which is important in therapy decisions [33]. All parameters can be defined based on the lateral cephalogram drafted on tracing paper, using the digital cephalometric analysis or both. Table 2 presents the most important cephalometric points according to known changes in class III malocclusion.

**Figure 4:**
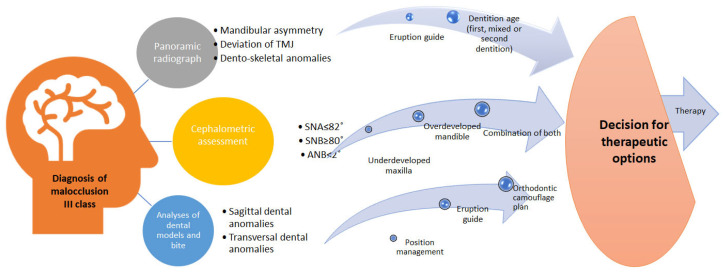
Diagnosis of malocclusion and therapy planning.

In conclusion, there are many therapeutic approaches for the treatment of class III malocclusions; however, there is still no common consensus regarding the most efficient device or method that could be used for class III malocclusion treatments (Figure 4). The aim of this review was to collectively analyse different treatments of class III malocclusion by the latest research articles and give a summation of treatment options. Also, we aimed to assess the therapy outcome and effectiveness in different severities of malocclusion, by analysing the craniofacial and dentoalveolar changes before and after therapy.

## 2. Discussion

Hereditary factors indicate that the degree of skeletal discrepancy can progress during a period of growth and development during puberty. This provides a therapeutic option that can destimulate condylar growth and mandibular skeletal overgrowth, such as chin cups, or stimulate right cross-bite and teeth position using an eruption guide expander. Still, if bad habits are the main reason that promote mandibular overgrowth, its removal is a priority using proprioceptive methods. If a diagnosis points to the underdevelopment of the maxilla, the main therapy goal is the expansion of maxilla growth, so that further mandibular growth can follow, using maxillary traction, face mask, or different surgical or nonsurgical palatal expanders.

Still, if occlusal discrepancy is diagnosed in adolescence, where the treatment approach for stimulation of maxilla expansion or destimulation of mandibular growth is limited or impossible, surgical therapy must be considered.

Therapy options can be divided into the following groups—orthopedic, orthodontic, and surgical. Orthopedic therapeutic options are chin cups, face masks, rapid palatal expansion, bone-anchored maxillary protraction, functional appliance, and eruption guides. Orthodontic therapy uses camouflage, fixed appliances (braces) and elastics, clear aligners, and temporary anchor devices. Surgical methods can be applied on both jaws as bimaxillary surgery; on maxilla as Le Fort I and surgical-assisted palatal expansion, mandible as condylectomy, sagittal split mandibular ramus osteotomy, bilateral sagittal split osteotomy, distraction osteogenesis, genioplasty, etc.

In this narrative review, an assessment was conducted based on the measured angles to review the success of the different therapy outcomes in malocclusion class III. The SNA, SNB, and ANB angles (cephalometric values) are the best to examine skeletal improvements [34]. Dentoalveolar improvements were also described, emphasizing the limitation of orthopedic therapy to change the skeletal discrepancy. Many other factors related to therapy success are described in certain subsections giving a broader picture about therapeutic approaches and considerations regarding it. With this review, we wanted to give the summation of the most current knowledge about therapy, limitations, and additional factors that can influence therapy with an aim of helping clinicians and researchers to focus on the specific approaches.

### 2.1. Orthopedic Therapy

#### 2.1.1. Eruption Guidance Appliances

Eruption guidance appliances (EGA) with differential occlusal thicknesses are generally used to treat class I and class II malocclusions, but these appliances are suggested to be used in the early phases of developing of malocclusion III. If developed, the class III malocclusion tends to worsen with age because the growth of the mandible exceeds the growth of the maxilla and anterior cross-bite favors such a situation. A new eruption guidance appliance has been developed to correct class III malocclusion in the early mixed dentition, anterior cross-bite with minimal skeletal discrepancy in a period eruption of permanent teeth, in approximately 20 months of therapy. The results showed a minimal effect on skeletal measurements over time, or compared to Frankel FRIII, similar effects in changes to the mandibular plane angle after treatment. Class III removable functional appliances used in skeletal class III discrepancies have limited effects, so a face mask should be considered as the first option [35,36].

#### 2.1.2. Chin Cups

A chin cup is a commonly used, non-invasive treatment that helps correct an underdeveloped mandible or excessive overbite, often in children and teenagers. A chin cup is a removable device that is typically made of plastic and metal consisting of a chin pad that rests on the chin and two side elastic bars that extend upwards to attach to the headgear or braces.

Singh et al. demonstrated the effectiveness of chin cups used as an early intervention can manage an anterior cross-bite and mandible prognathism, in a 12 year old boy, using braces and fixed orthodontics appliances to fix anterior cross-bite and to establish normal overjet and overbite in front, and chin cup only to limit mandibular growth [37].

Chin cup usage has faced some controversy due to side-effects causing the condyle harm by decreasing their growth, leaving consequences in the future. Some articles have also described the restriction of mandibular growth and decrease in the gonial angle and posterior movement of the B point and pogonion [38]. Still, others have presented that using a chin cup only during the night in 2.5 years can decrease SNB for a mean of 1.50 but with no change in gonial angle and mandible length. It is noted that the success of therapy may vary from the force appliance and the duration of wearing the cup during the day [39]. A study that followed condylar position and volume using CBCT to accurately proper volumetric parameters found no effect to mandibular dimension using a chin cup, and also that the major action of the retroactive force on the chin cup oriented onto the condylar position is associated with compression of the joint spaces [40].

#### 2.1.3. Dental Circuit Breaker

A dental circuit breaker is an interceptive method used in the early stages as a strategic and prevention approach in treatment of malocclusions, usually when smaller discrepancy exists and one of the main reasons of using this method are treating the lateral or posterior cross-bites, underdeveloped maxilla or as facilitators for other orthodontic treatment, by widening the palate, and can be used in different age. It is described that the combined effect of face mask and dental circuit breaker; Alt-RAMEC and face mask; and disjunction and face mask had a significant increase in SNA, SNB and ANB angle at the end of the treatment in patients with class III malocclusion [34].

#### 2.1.4. Face Masks

A face mask, also called reverse headgear, is the appliance used in orthodontics to treat the under-bites by pulling out the maxilla in a growing patient so it can match the growth of the mandible. There are also multi-adjustable face masks that can increase comfort for the patients. Among the various approaches for anterior cross-bite correction, face mask therapy is considered the preferred treatment.

Face mask effectiveness combined with anchorage of a palatal expander was evaluated in Vietnamese study to see the impact on skeletal class III, where two groups of children divided into the pre-puberty and mid-puberty group were followed. At the end of treatment, the SNA angle increased by 2.2 degrees, while SNB angle decreased by 1.2 degrees. The increase in the SNA angle was slightly higher in the pre-puberty group, while the SNB angle decreased more in the mid-puberty group. In both groups, the ANB angle increased by roughly 3.0 degrees, and more in the pre-puberty group. Overall, the treatment outcome was successful in both groups, with a higher percentage in the pre-puberty group [41].

In the study by Li et al., patients of different dentition periods were followed after the maxillary retraction with malocclusion type III combined maxillary insufficiency growth, and by the results, the dentition did not influence the success in any therapy outcome. They were treated with a Delaire face mask, and both skeletal and dentoalveolar changes were observed. A study recommended that therapy should start at C2 cervical vertebra maturation stage, after which the SNA angle increases by 1.80 degrees, the SNB decreases by 1.28 degrees, and the ANB increases by 3.09 degrees [42]. The compliance-free, NET3-corrector, and conventional RME-face mask were compared in their effectiveness of treating the malocclusion type III in patients aged 8–14 at stage 4 cervical maturation, without any previous treatment of malocclusion in a 3-week duration. The study concluded that the NET3-corrector was superior in correcting the maxillary insufficiency and dentoalveolar position of inferior incisors, where the measured angle changed as follows: SNA increased by 3.14 degrees, the SNB by 0.14 degrees, and the ANB by 3.02 degrees, comparing the measured angles before and after treatment. Advantages of NET3-connectors are that they need only permanent molars and two palatal screws for anchorage, so the advantage is that it can be used before the eruption of the premolars and canines. Also, the screws positioned in the palate show a high success rate and there is no possibility for root damage [43].

A comparative clinical study investigated two maxillary protraction protocols, including bone anchors and a Delaire-type face mask, proving the protractor being more advanced in changes of skeletal deformity in the short-term, especially in SNA angle, with 2.3 degrees change in the bone protractor compared with 1.2 degrees in the face mask. Also, the benefit of bone anchors is that they are easily integrated into daily routine, because elastics can be worn in public [44].

In the research conducted among orthodontists, the opinion about treating malocclusion III concluded that the vast majority used rapid palatal expander and face masks in duration from 9 to 12 months, where specialists of orthodontics preferred a Petit face mask, with an average age of patients being 5 to 8 years old [45].

#### 2.1.5. Reverse Twin Block

Reverse twin block (RTB) is a variation of the traditional twin block appliance proposed by Clark, used for quick correction of developing malocclusion type III where the occlusal inclined planes are reversed, and inclined planes are set to 70 degrees to the occlusal plane, with bite blocks being over the lower molars and upper deciduous molars or premolars with sagittal screws to advance the upper incisors. The use of the reverse twin block is appropriate for younger patients, in early mixed dentition, when the development of malocclusion class III can be directed through growth modifications. Mild mandibular prognathism with a normal or mild maxillary retrognathism are the ideal skeletal indicators for the use of RTB. Primarily, the modification is limited to the dentoalveolar area and slight skeletal change (SNA and ANB angles increased by 2 degrees, while in the 2-year follow period there was additional change, even in SNB angle) [46]. In a comparable study, the effectiveness of the twin block with lip pads and fixed rapid maxillary expansion and a face mask with rapid palatal expansion was assessed. No significant difference was noted between those two methods, but the ANB increased by 3 degrees, SNA by 2 degrees, and SNB decreased by 1 degree. The limitations of this method are the lack of follow-up measurements of skeletal class progress, making comparability difficult [47]. The case report study showed that RTB treatment confirmed its primarily dentoalveolar change, promoting the normal position between upper and lower teeth, where upper teeth proclined by 3.0 degrees and lower retroclined by 3.0 degrees. Considering angles, the SNA angle stayed unchanged pre- and post-treatment, and SNB increased by 2.0 degrees and ANB decreased by 2.0 degrees [48].

#### 2.1.6. Maxillary Expanders and Palatal Disjunction

Rapid maxillary expansion (RME) is a traditional appliance used to correct transverse discrepancies in patients whose midpalatal sutures are not fully closed. A very popular protocol for treatment of malocclusion III is Alternative Rapid Maxillary Expansion and Constriction (Alt-RAMEC) was introduced by Liou [49]. Using this protocol, the maxillae will be enlarged by 1 mm per day for 7 days with total of 7 mm enlargement, and then the 1 mm screw is closed. In other weeks, the screw of the expansion device is turned on for one week and then closed for one week, completing the Alt-RAMEC protocol at the end of the nine-week process. This procedure seemed to be more promising in the orthodontics than single rapid palatal expansion and maxillary protraction, with a three and two times greater anterior displacement of the maxilla.

On the other hand, the transverse separation of the maxilla, through rapid palatal disjunction (RPD), is considered as one of the most impressive orthopedic procedures. Disjunction is an orthopedic procedure that consists of separating and disjoining the two segments that form the upper jaw by means of force, allowing new bone formation in the space that remains free between the edges of the separation, being a suture modeling therapeutic mid palatine and middle third of the face. This procedure can be used in any skeletal malocclusion, I, II, and III.

The combined effect of a face mask and Alt-RAMEC protocol also showed an increase in SNA angle between 1.74 and 4.10 degrees, decrease in SNB angle between 0.18 and 2.38 degrees, and an increase in ANB angle between 3.04 and 4.48 degrees. Then, the combination of disjunction and facial mask showed an increase in SNA angle between 0.22 and 1.64 degrees, decrease in SNB angle between 0.01 and 1.32 degrees, and increase in ANB angle between 0.61 and 2.65 degrees [34].

In the study by Guo et al., protocol Alt-RAMEC showed positive outcomes after six weeks with on and off activation and deactivation of the expanders. It was shown that tonsil hypertrophy can influence malocclusion III, and patients after tonsillectomy had normalization of dentofacial growth [50].

### 2.2. Orthodontic Therapy

Maxillary deficiency and diagnosed class III malocclusion have been treated with a maxillary skeletal expander (MSE) with four mini-implants, with the aim of correcting the skeletal class and dentoalveolar problem of bilateral cross-bite and crowding. There were changes in SNA, SNB, and ANB angles by +1.5, −1.1, and +2.6 degrees, consequently [51]. Temporary anchorage devices (TADs) are generally mini-screws placed in either alveolar or extra-alveolar bone for the purpose of providing orthodontic anchorage. The hallmark of this device is its intended removal once it has completed its function in the treatment regimen. Using anchorage devices in mandibular arch distalization is also one more option for treating class III without surgery. By the results from Setvaji’s review, the SNB and ANB angles in TADs changed very little or did not change at all, but dentoalveolar changes, like molar distalization and distal tipping, were more obvious. Then, miniplates showed greater distalization compared to mini-screws, while interradicular mini-screws exhibited the least distal tipping. Retromolar mini-screws showed less molar and incisor distal tipping than interradicular mini-screws while SNA and ANB decreased more in retromolar mini-screws [52].

The therapy with miniplates is a method that can help midface growth in younger patients with an underdeveloped maxilla. They are modified osteosynthesis fixation devices, and their fixation screws are placed apical to the roots so they do not affect the movement of teeth. They are also placed through attached gingiva, so the placement is very stable and also its position tends to be close to the attachment unit placed on the dental arch. Due to their stability, they can allow the much higher forces necessary for orthodontic movement, but they do need to be placed and removed by oral surgeon under the local anesthesia. Over 90% of used miniplates were successful and the most common issue was swelling of the surrounding tissue [53]. Combining the Delaire-type face mask with miniplates via the elastic force, the remarkable advancement in the middle face was fulfilled. Miniplates were positioned on the lateral nasal wall of the maxilla and provided perfect stability during the therapy protocol. This protocol was a good option for late mix dentition and provided significant changes in SNA, SNB, and especially, ANB angles [54]. Additional research with a higher number of participants could confirm the effectiveness of the proposed therapeutic option. Mini-screws and miniplates were also used with same purpose—to help in maxillary protraction, and they were increasing ANB angle by 5 degrees and SNB by 1.9 degrees [55,56].

Micro-implant-assisted rapid palatal expansion (MARPE) has shown high success rates for transverse maxillary expansion in late adolescents and adults, presenting a viable alternative to surgically assisted rapid palatal expansion (SARPE). This case report by Chung et al. included malocclusion with skeletal position of class III; both presented anterior and posterior cross-bite and moderate dental crowding and were treated with MARPE for the upper jaw and temporarily anchorage devices for the lower jaw. The SNB angle decreased by 2 degrees and the ANB angle increased by 2 degrees, reducing the severity of class III, retaining the results after one year follow-up [57]. The combination of MARPE and lingual appliances have proven to be a viable treatment alternative for adult class III malocclusion with maxillary constriction, but with less success in the skeletal change, considering SNA, SNB, and ANB angles [58].

In cases of maxillary deficiency, the palatal–plate face mask combination (a mini-screw-retained palatal C-shaped plate and face mask) showed great results in patients with an early mix dentition. The class III skeletal malocclusion was corrected into class I with significant change in angles before and after therapy. The ANB angle increased by 4.5 degrees, SNA angle by 3.1 degrees, and the SNB angle decreased by 1.4 degrees at the end of the therapy. This technique is not recommended for patients with transversal deficiency [59]. In older patients, the treatment of malocclusion III gets more complex, but limited at the same time, and sometimes the only option is orthognathic surgery. There are several options before bimaxillary surgery but usually the most accepted therapeutic option for patients has been orthodontic camouflage.

While surgery often provides definitive results for severe cases, orthodontic camouflage is a viable alternative for managing mild to moderate skeletal discrepancies in adults because the patients are often afraid of the severity of intervention, and post-operative recovery. The aim of orthodontic camouflage is to enable the displacing of teeth relative to their supporting bone to compensate for an underlying jaw discrepancy while maximally attaining acceptable occlusion, function, and esthetics.

The orthodontic camouflage can be performed with and without tooth extraction.

One plan of orthodontic camouflage involves fixed orthodontic appliances (braces) in both jaws with class III intermaxillary elastics to encourage movement of maxilla forward and mandible backward. After this stage, the fixed retainers are used in both jaws to retain results. The results were maintained after three months at regular follow-ups in a non-growing patient [60]. A similar plan was used in other case reports which resulted in a decrease in the SNB angle of 7 degrees, and an increase in the ANB by 7 degrees [61]. Dental camouflage can also be applied in a growing patient. The treatment plan consists of distalization of lower canines, retraction of the lower anterior segment, and class III ligatures. There were changes in angles, increase in SNA and SNB by 2 and 3 degrees, and a decrease in ANB angle by 1 degree [62]. Class III with a bilateral cross-bite and anterior open bite had an approach with non-extraction camouflage with the use of palatal mini-implant, transpalatal arch, and cantilever TMA wire, with SNA, SNB, and ANB angles increasing by 0.7, 0.4, and 0.3 degrees at the end of therapy [63]. The camouflage can be used in both growing and non-growing patients, and it seems to have good results at the end of both cases, keeping this treatment option open for patients that are not ready for the more radical procedures.

Extraction camouflage with extraction of premolars of upper and lower jaw, combined with mini-screws skeletal anchorage was used for treating a severe class III malocclusion. The outcome was visible on the dentoalveolar segment where the anterior cross-bite and crowded teeth were fixed. The indicators of skeletal improvement had minor change in the case report with SNA, SNB, and ANB angles changing by +0.3, −0.2, and +0.5 degrees [64].

### 2.3. Surgical Therapy

Orthognathic surgery for dealing with moderate to severe cases of type III malocclusion is considered as the standard measure with change in both skeletal discrepancy and dentoalveolar misalignment. It was speculated that this procedure may be intertwined by morphological changes in condyle, disc, and fossa in previously published studies, as well as muscle change. Studies dealing with analysis of condylar angle noted that this angle was higher in skeletal class II than skeletal class III, but it changes post-surgically in both. Although changes were more obvious in malocclusion type II, no significant difference was found when comparing with class III except that condyle resorption tends to be more pronounced in class II [65]. This indicates that resorption can be specific for certain skeletal patterns and that is related to the surgical extension, so this must be an important factor that should be known prior to the decision of the operation. Joint space also increases after surgery and retains stability after 9 months [66]. Some studies showed an increase in temporomandibular symptoms after surgery, which was also a controversial result in other studies where the TMJ clicking was reduced, and the surgery was not proven to be a significant factor for development of symptoms [65,67].

Liu et al. compared results of orthodontic camouflage and orthognathic surgery measurements of angles SNA, SNB, ANB, and influence on mandibular anterior teeth position in class III malocclusion. The group with orthodontic camouflage followed the protocol with transmission straight-wire appliances and with four teeth extracted or without extraction. The group with orthognathic surgery used maxillary advancement, mandibular setback, and genioplasty along with extraction of four, and more commonly two, teeth. Orthodontic camouflage poorly changed the SNA and SNB angles but decreased the ANB angle by 1.2 degrees [68]. In a similar investigation, the camouflage could be helpful in managing mild to moderate cases of malocclusion class III while tilting the lower teeth more backward, giving satisfying esthetics and better position between root and bone. Movement of lower teeth was carefully investigated because the movement of lower incisors causes alveolar bone loss. On the other hand, the orthognathic surgery increased SNA and ANB angles by 2.91 and 5.72 degrees, and decreased SNB by 2.83 degrees [68]. It significantly improved the skeletal and dentoalveolar characteristics of malocclusion, while giving the overall esthetic of the face. This indicates that orthognathic surgery is a more effective therapy for overall change, especially in more difficult class III malocclusion like real mandibular prognathism. The SNA angle decreased by 0.6 degrees and the SNB angle by 2.9 degrees when compared the pre- and post-surgery results after one year of bi-maxillary surgery, while the length of the ramus decreased and width increased [69]. The surgery demands a more invasive approach, strict and long period of post-treatment recovery so many patients, especially the younger ones, chose camouflage over surgery if possible. In the long-term, the esthetic after the surgery treatment of patients with true mandibular prognathism was proven to be retained considering following smile parameters—buccal corridor, upper lip height, and smile index [70].

Bilateral condylectomy is proposed for individuals with posterior vertical excess caused by condyle hyperplasia or acromegaly. The intervention consists of condyle resection guided by the proper assessment of the mandibular angle and followed by the Le Fort I osteotomy for placing the upper and lower jaws into the new position. The results of this intervention become stable after approximately 20 months. Due to activation of pterygo-masseteric sling, the formation of new bone can happen due to intense bone remodeling which can result in an additional intervention—basilar bone resection. It was noted that one year after the bi-maxillary surgery, the masseter and medial pterygoid muscles increased their length by 1 to 2 mm [65,68]. Articles indicate that bilateral condylectomy may be a good alternative to the bimaxillary surgery and adds to the therapeutic options for the correction of class III malocclusion [71].

Dealing with a malocclusion class III on the individual level can be very challenging. Due to a noticeable outlook of the individual’s front face and profile, they develop a lot of insecurities which, if these persist, may affect the person on many levels. It has been proven that individuals with this skeletal class may be dealing with a problem with low self-esteem and anxiety. A very important part of therapy success of class III malocclusion is not only bringing the function, esthetic, and occlusion to the proper levels, but also to restore the psychological well-being. So, new articles are starting to focus on this component and trying to enlighten the importance of the well-being of the patients. In a recent article, evidence points out that there was an improvement in self-esteem, sensitivity to criticism, and social anxiety after the surgery; but still, it was higher compared to the individuals without malocclusion. An important part of the therapy is a correct approach to those individuals and pointing out the more realistic expectations that will occur after surgery [72].

**Figure 5:**
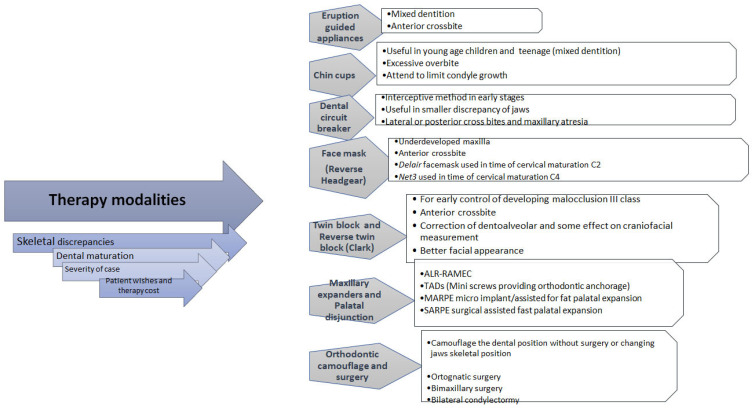
Therapy modalities for treatment of malocclusion III class.

One of the main limitations of articles dealing with therapeutic options is that they do not provide information about long-term outcomes. There are very few studies that showed that many of therapeutic options, which are not radical, do not retain the results for a long time. The therapeutic success was high at the end of therapy, but over time, the measured angels tended to change. The necessity for longer follow-up times should be very important to be considered for future studies [73,74]. Patient compliance is crucial in therapy success due to the required high patient cooperation which can limit success. As some orthodontic patients are underage, and their understanding about the function of the therapy is limited, this aspect is now redirected to the parent, which also can be challenging. It is very difficult to maintain the long-lasting check-ups with either underage persons or adults due to different factors—from life situations to certain global factors, such as pandemics. The lack of original articles focusing to the new therapeutic options for malocclusion class III is also very concerning, and the newer approaches are mainly described through case reports which are not based on a very strong body of evidence like original or systematic articles (this can be seen in the Table 3). Thus, the therapeutic approaches may become overwhelming nowadays. Original articles with new approaches and a longer period of check-up must be imperative in future studies (Figure 5).

## 3. Conclusions

A variety of orthopedic and orthodontic treatments, and duration of therapy protocols make it harder for clinicians to choose which is the best as long-term malocclusion solution. There is a limiting factor for choosing the right therapy option. Orthopedic appliances can influence growth, but outcomes can be very variable and less effective in late adolescence or adulthood. Also, their use has more effect in early childhood and mixed dentition, during tooth eruption, and in cases with no skeletal discrepancy. Eruption-guided appliances can be useful in anterior cross-bite, while the chin cups can only limit further growth of the mandible, but with no significant skeletal changes. If the malocclusion is connected with skeletal discrepancy, the face mask is the first choice as orthopedic therapy. If the maxilla is undeveloped, different types of palatal expanders can be used in transversal jaw expansion. A different approach must provide a stable anchor for force application to be effective for expansion in the short-term, but it should not be robust. Today, there is a problem of delayed start of the expansion, and waiting until permanent dentition. Anchorage of forces during palatal suture expansion may be secured by plates, mini-implants or mini-screws. Still, following the esthetic standards, the bulkiness of those appliances makes them unwanted to wear during the period of childhood and puberty. Also, this therapy has limited possibilities, which makes the fixed orthodontic appliance with braces and elastic traction the most applied therapy modalities today. The malocclusion with major skeletal discrepancy must be considered for surgical therapy, as the only solution for achieving facial symmetry and face esthetic. In those cases, the orthodontic appliance is used only to prevent the further malocclusion progression or can be useful in preoperative teeth placement to surgically plan position.

Most studies that we examined during our research simulated more than one therapy modality in class III, for different dentition types and time periods. So, further new studies with similarity in research groups and therapy approaches must be conducted to understand long-term therapy approaches and their success. Also, further research can follow the benefits of different orthodontic appliances before surgery and a period of preoperative therapy.

## Figures and Tables

**Table 1:** The classification of malocclusion by Steiner.

Steiner Analysis		
Skeletal analysis	Dental analysis	Soft tissue analiysis
SNA SNB ANB Occlusal plane Mandibular plane	Maxillary Incisor Position (U1-NA) angle and distance (2 measurements) Mandibular Incisor Position (L1-NB) angle and distance (2 measurements) Interincisal Angle Lower Incisor to Chin	S-line

**Table 2:** Cephalometric analysis class III patients according to anatomic changes.

Class III Characteristics	Cephalometric Parameters
Maxillary deficiency	SNA ≤ 82°
Mandibular prognathism	SNB ≥ 80° ANB < 2°
Maxillary incisor protrusion	Maxillary incisor position U1-NA angle (axial upper incisors relation to NA line) > 22° protrusion < 22° retrusion U1-NA distance (NA distance to the most medial surface of upper incisors) > 4 mm protrusion < 4 mm retrusion
Mandibular incisor retrusion	Mandibular incisor position L1-NB angle (axial lower incisors relation to NB line) > 25° protrusion < 25° retrusion L1-NB distance (NB distance to the most medial surface of lower incisors) > 4 mm protrusion < 4 mm retrusion
Difference in mandibular effective length	GoGn-SN > 32° vertical growth GoGn-SN < 32° horizontal growth pattern
Difference in facial height	According to symmetry of facial a thirds Hairline–Glabella; Glabela–Subnasale; Subnasale–Mention

**Table 3:** Summation table of characteristics of the included studies (year, therapy approach, article type, number of participants, duration of therapy, and mean age) with cephalometric measures (SNA, SNB, and ANB), and assessed by evidence strength.

		Characteristics of Included Studies					Cephalometric Measures						Evidence Strength
Authors	Year	Therapy Approach	Article Type	Number of Participants	Duration of Therapy (Months)	Age (Years, Mean)	SNA°		SNB°		ANB°		
							Before	After	Before	After	Before	After	
Otel et al. [34]	2025	Dental circuit breaker and face mask	Systematic Review Meta-analysis	20–40	6	6–12	NA	NA	NA	NA	NA	NA	Strong
		Alt-RAMEC and face mask					NA	NA	NA	NA	NA	NA	
							NA	NA	NA	NA	NA	NA	
		Disjunction and face mask											
Velasquez et al. [35]	2024	Eruption guidance appliance	Original article	44	20	7.6	81.23	81.19	80.05	80.57	1.20	0.62	Moderate
Pellegrino et al. [36]	2020	Eruption guidance appliance	Case report	1	7	5.5	74.0	NA	73.0	NA	1.0	NA	Mild
Singh et al. [37]	2024	Chin cup	Case report	1	25	12	NA	NA	NA	NA	NA	NA	Weak
Chang et al. [38]	2005	Chin cup	Original article	20	17	9.5	NA	NA	NA	NA	NA	NA	Moderate
Barrett et al. [39]	2010	Chin cup Chin cup with quad helix	Original article	26	30	8.5	79.4	78.5	80.0	78.7	−0.7	0.4	Moderate
Husson et al. [40]	2023	Chin cup with bonded maxillary bite block	Randomized control study	38	16	6.6	NA	NA	NA	NA	NA	NA	Strong
Ly et al. [41]	2024	Face mask and anchorage of a palatal expander	Original article	31	3	7–12	79.37	81.5	81.39	80.26	−1.98	1.37	Strong
Li et al. [42]	2024	Delaire face mask with maxillary expander	Retrospective original study	97	NA	5.91	78.29	80.01	79.36	78.29	−1.06	3.85	Strong
						9.05	77.93	79.71	80.19	79.48	−2.27	4.96	
						10.60	76.76	78.58	80.73	79.33	−3.96	7.15	
Tarraf et al. [43]	2025	Net3 corrector RME-face mask combination	Retrospective original study	20	10.5	11.14	79.40	82.54	80.47	80.62	−1.10	1.92	Strong
					12		77.85	78.90	79.73	78.77	−1.90	0.12	
Tabellion et al. [44]	2024	Bone anchors and face mask	Original article cephalometric analysis	31	13.5	11.0	80.13	82.43	81.63	82.34	−1.51	0.08	Moderate
					10.0	6.74	80.30	81.52	80.12	79.31	0.16	2.20	
Franchi et al. [45]	2024	Face mask	Web-based questionnaire	151	NA	NA	NA	NA	NA	NA	NA	NA	Mild
Mittal et al. [46]	2017	Reverse twin block	Case report	1	10	11	80.0	82.0	84.0	84.0	−4.0	−2.0	Moderate
Minase et al. [47]	2019	Reverse twin block	Original article	39	9	10.17	79.15	81.15	82.23	81.15	−3.07	0	Strong
		Face mask					78.92	80.23	81.46	80.73	−2.53	−0.50	
Pandey et al. [48]	2014	Reverse twin block	Case report	1	8	10	82.0	82.0	80.0	82.0	2.0	0	Mild
Liou et al. [49]	2005	Alt-RAMEC	Original article	26	6	10.5	NA	NA	NA	NA	NA	NA	Moderate
Guo et al. [50]	2024	Alt-RAMEC/PFM	Original	96	1.5	5.0	NA	NA	NA	NA	NA	NA	Moderate
Wang et al. [51]	2024	Maxillary skeletal expander	Case report	1	39	15	73.7	75.2	76.0	74.9	−2.3	0.3	Moderate
Setvaji et al. [52]	2024	Skeletal Temporary Anchorage Device	Systematic Review	NA	6–12	NA	NA	NA	NA	NA	NA	NA	Strong
Corenelis et al. [53]	2008	Miniplates for skeletal temporary anchorage	Original article	97	NA	24.0	NA	NA	NA	NA	NA	NA	Moderate
Kircelli et al. [54]	2008	Face mask with skeletal anchorage	Pilot	6	6–8	11.8	75.0	78.7	80.3	78.0	−5.2	0.9	Weak
Souza et al. [55]	2020	Maxillary protraction with mini-screws	Case report	1	16	10.0	78.0	82.0	83.0	82.0	−5.0	0	Mild
Eid et al. [56]	2016	Maxillary protraction with miniplates	Original	10	12	10.05	77.05	76.75	80.0	81.9	−2.7	1.95	Moderate
Chang et al. [57]	2024	Mini-screw palatal expansion	Case report	1	10	21.0	81.0	81.0	83.5	81.5	−2.5	−0.5	Mild
Nguyen et al. [58]	2024	Mini-screw palatal expansion	Case report	1	11	29.0	79.1	79.3	80.8	80.5	−1.7	−1.2	Mild
Elsaharty et al. [59]	2024	Mini-screw Palatal C-shaped plate face mask	Original article	16	6	8.0	77.14	80.27	80.45	79.07	−3.3	1.8	Moderate
Ginali et al. [60]	2024	Orthodontic camouflage	Case report	1	20	19.0	NA	NA	NA	NA	NA	NA	Weak
Nyakale et al. [61]	2025	Orthodontic camouflage	Case report	1	15	22.0	82.0	82.0	93.0	86	−11.0	−4.0	Mild
Mazzini et al. [62]	2017	Orthodontic camouflage	Case report	1	17	13.0	84.0	87.0	88.0	90	−4.0	−3.0	Mild
Popov et al. [63]	2024	Skeletal anchorage	Case report	1	23	26.0	78.8	79.5	77.7	78.1	1.1	1.4	Mild
Zhang et al. [64]	2025	Extraction camouflage	Case report	1	31	18.0	84.9	85.2	85.3	85.1	−0.4	0.1	Mild
Faria-Teixeira et al. [65]	2025	Orthodontic surgery	Systematic review	NA	NA	NA	NA	NA	NA	NA	NA	NA	Strong
Kuehle et al. [66]	2016	High oblique sagittal split osteotomy	Original article	50	9	26.3	NA	NA	NA	NA	NA	NA	Moderate
Zhai et al. [67]	2020	Orthognathic surgery	Original article	182	NA	22.5	NA	NA	NA	NA	NA	NA	Strong
Liu et al. [68]	2024	Orthodontic camouflage	Original article, retrospective	40 in each group	NA	18.42	81.09	81.82	83.78	83.58	−2.69	−1.76	Strong
		Orthognathic surgery				21.22	80.94	83.85	84.22	81.39	−3.26	2.46	
Takayama et al. [69]	2019	Bimaxillary surgery	Original article,	42	24	NA	81.5	80.9	82.6	79.7	NA	NA	Moderate
Chiang et al. [70]	2024	Orthognathic surgery	Original article, retrospective	34	NA	21.6	NA	NA	NA	NA	NA	NA	Moderate
Derquenne et al. [71]	2024	Bilatteral condilectomy	Short communication	NA	NA	NA	NA	NA	NA	NA	NA	NA	Mild
Wang et al. [72]	2024	Bimaxillary surgery	Original article, observational	205	NA	21.9	NA	NA	NA	NA	NA	NA	Strong
